# Supplementary material for: Characterization and Immunological Activity of Exopolysaccharide from Lacticaseibacillus paracasei GL1 Isolated from Tibetan Kefir Grains
Source: Foods. 2022 Oct 23;11(21):3330. doi: 10.3390/foods11213330 (PMC9656536; doi:10.3390/foods11213330)
Supplement: Supplementary file 1 [file foods-11-03330-s001.zip › foods-1961877-supplementary.pdf]

**Table S1**. The sequence of primers

| Primer | | Sequence |
|---|---|---|
| 16S rDNA | Forward | AGAGTTTGATCCTGGCTCAG |
| | Reverse | TACGGYTACCTTGTTACGACTT |
| TNF-$\alpha$ | Forward | GGGGATTATGGCTCAGGGTC |
| | Reverse | CGAGGCTCCAGTGAATTCGG |
| IL-1β | Forward | TCCAGGATGAGGACATGAGCAC |
| | Reverse | GAACGTCACACACCAGCAGGTTA |
| iNOS | Forward | CAAGCTGAACTTGAGCGAGGA |
| | Reverse | TTTACTCAGTGCCAGAAGCTGGA |
| β-actin | Forward | CATCCGTAAAGACCTCTATGCCAA |
| | Reverse | ATGGAGCCACCGATCCACA |
